# A cohort study of antihyperglycemic medication exposure and survival in patients with gastric cancer

**DOI:** 10.18632/aging.102245

**Published:** 2019-09-13

**Authors:** Audrius Dulskas, Ausvydas Patasius, Donata Linkeviciute-Ulinskiene, Lina Zabuliene, Giedre Smailyte

**Affiliations:** 1 Department of Abdominal and General Surgery and Oncology, National Cancer Institute, Vilnius LT-08406, Lithuania; 2 University of Applied Sciences, Faculty of Health Care, Vilnius ELT-08303, Lithuania; 3 Institute of Clinical Medicine, Faculty of Medicine, Vilnius University, Vilnius LT-03101, Lithuania; 4 Laboratory of Cancer Epidemiology, National Cancer Institute, Vilnius LT-08406, Lithuania; 5 Institute of Health Sciences, Faculty of Medicine, Vilnius University, Vilnius LT-03101, Lithuania; 6 Institute of Biomedical Sciences, Faculty of Medicine, Vilnius University, Vilnius LT-03101, Lithuania

**Keywords:** gastric cancer, metformin, diabetes, population-based study, antihyperglycemic

## Abstract

Objective: We aimed to estimate survival in gastric cancer patients with type 2 diabetes mellitus (T2DM) using different antihyperglycemic medication.

Methods: Patients with gastric cancer and diabetes between 2003-2013 were identified form The Lithuanian Cancer Registry and The National Health Insurance Fund database. Cohort members were classified into five groups: four groups of T2DM patients according to treatment: metformin users; metformin and other medication users; sulphonylurea users; insulin and other medication users; and non-diabetic group. Kaplan-Meier survival analysis and Cox proportional hazards models were used to estimate gastric cancer-specific survival and overall survival.

Results: 8423 patients met eligibility criteria. Survival analysis showed no differences in gastric cancer-specific survival between non-diabetic and diabetic patient groups. Better survival was observed in the groups of patients using antihyperglycemic medication combinations with metformin, metformin alone or insulin. Lowest survival was observed in diabetic patients who were sulphonylurea users. Survival analysis comparing overall survival between non-diabetic and diabetic patients (p = 0.89) showed no evidence of survival difference between groups and survival differences between antihyperglycemic medication user groups were of borderline significance (p = 0.052).

Conclusions: Antihyperglycemic medication use was not associated with a significant effect on survival in patients with gastric cancer and T2DM.

## INTRODUCTION

Gastric cancer is a deadly disease which is responsible for over 1,000,000 new cases in 2018 worldwide and an estimated 783,000 deaths (equating to 1 in every 12 deaths globally), making it the fifth most frequently diagnosed cancer and the third leading cause of cancer death [1].

The International Diabetes Federation (IDF) estimated that 1 in 10 adults aged 20–79 years (425 million of adults) had diabetes mellitus globally in 2017 [2]. Obesity and T2DM have been linked to many types of cancer, including gastric cancer. This association has primarily been attributed to insulin resistance and cluster factors of metabolic syndrome, which also play an additive carcinogenic role [3].

T2DM can be treated with metformin, sulfonylurea, insulin or other classes of antihyperglycemic medication. Metformin is the most common first-line treatment for T2DM. Besides the glucose-lowering effect, metformin interferes with carcinogenesis through indirect and direct mechanisms. Hyperinsulinemia increases cancer risk in healthy subjects and can partly explain the obesity-cancer risk association in many organ sites, including the colon. Hyperglycemia is also a risk factor for gastric and other cancer sites [4, 5]. However, direct antitumor mechanisms for metformin have been implicated because preclinical studies have shown that metformin can inhibit the growth of all cancer cells (including gastric cancer cells) *in vitro* and *in vivo* [6, 7]. Several mechanisms have been suggested, for example, it reduces insulin resistance and suppresses the mammalian target of rapamycin (mTOR) by activating the liver kinase B1 (LKB1) dependent adenosine 5′-monophosphate-activated protein kinase (AMPK) pathway. It may also inhibit protein synthesis, and unfolded protein response, activate the immune system, eradicate cancer stem cells, suppress cell proliferation or induce cell cycle arrest and/or apoptosis and many others protective mechanism [8]. By lowering elevated insulin levels it reduces activation of the PI3K-mTOR pathway, which is responsible for cell survival and proliferation in cancer [5]. Another possible mechanism of action was recently described by Valaee et al. [9]. Research showed, that metformin inhibits epithelial-mesenchymal transition, which is one of the main agents contributing to tumour spread, in human gastric cell line. We found that metformin has a positive effect on prostate and colorectal cancer by decreasing the incidence risk and prolonging the overall and cancer specific survival [10–12].

Previous studies have shown that, among antihyperglycemic medication, insulin and sulfonylurea may increase the risk of cancer by interacting with insulin and insulin like growth factor 1 (IGF-1) receptor signalling, which enhances proliferation and carcinogenesis [13, 14]. Insulin is a growth factor and has metabolic and mitogenic effects [15, 16]. Hyperinsulinemia, especially in the presence of insulin resistance, may promote cancer cell growth either through insulin receptor or IGF-1 receptor, or via increased bioavailability of free IGF-1 by inhibiting the expression of IGF binding proteins. These pathways have also been shown in gastric cancer cell lines both in vitro and in vivo studies [citavimas].

Gastric cancer and antihyperglycemic medication (mainly metformin) associations have been analysed in less than 20 studies [17–28]. Previous studies showed that diabetes decreases cancer survival and metformin has a protective effect [29–32]. However, there are only three studies on T2DM and the prognosis of gastric cancer [19, 33, 34]. No other study so far has assessed the effect of few different antidiabetic medications on gastric cancer survival.

The main objective of our study was to evaluate the effect of antidiabetic therapy on survival of patients with gastric cancer.

## RESULTS

**Figure 1:**
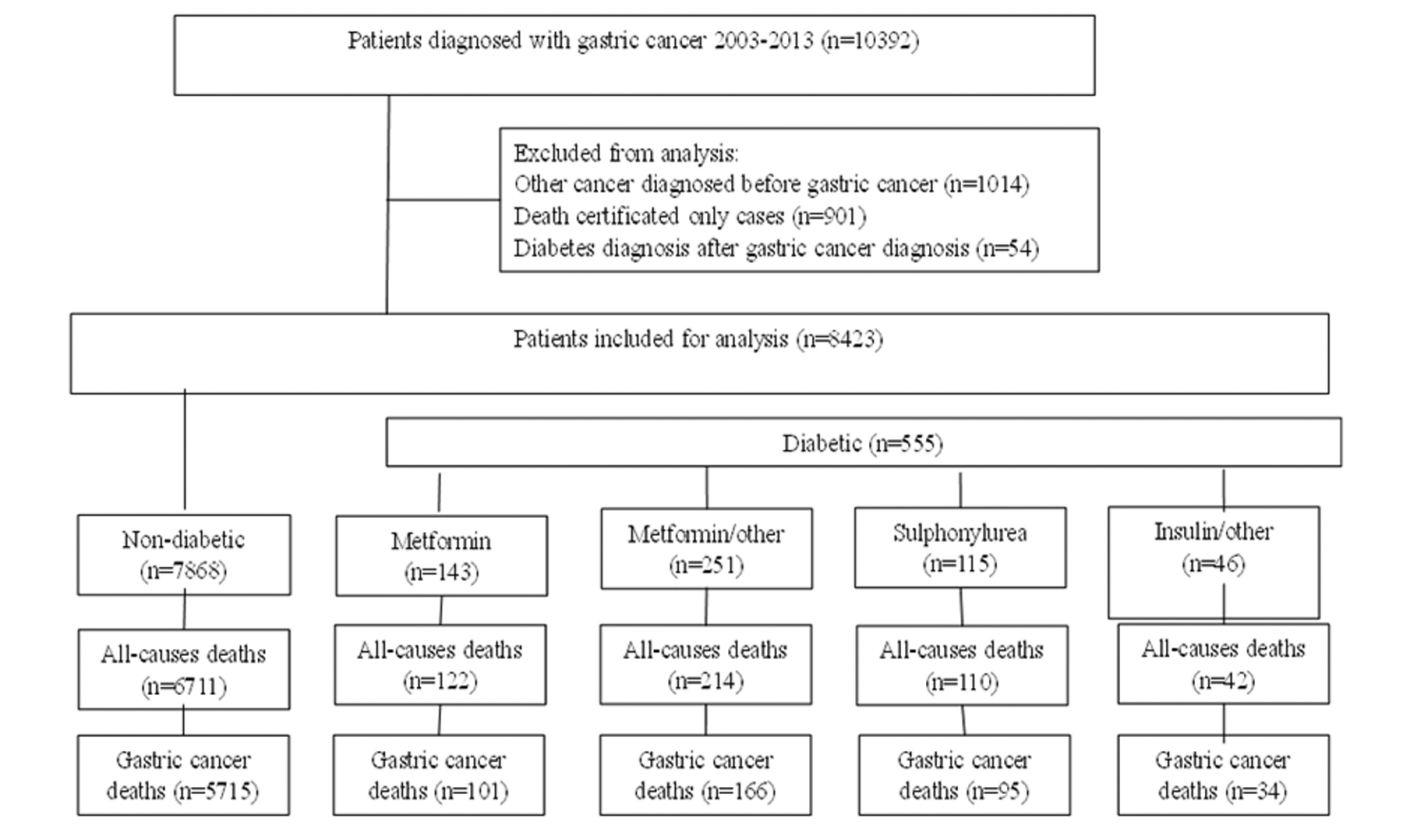
**Study flow chart of gastric cancer patients with type 2 diabetes undergoing medical treatment and without.**

After excluding patients with diabetes diagnosis after gastric cancer (N=54), patients with other cancer diagnosis before gastric cancer (N = 1014) and patients with cancer diagnosis at the day of death (DCO cases) (N = 901), there were 8423 patients who met eligibility criteria for this analysis, including 555 (6.59 %) with pre-existing T2DM and 7868 patients without diabetes (Figure 1). During follow-up there were 7199 deaths including 6111 from gastric cancer. The study group included 58.38 % male and 41.62 % female gastric cancer patients. At the time of diagnosis 27.09 % of patients were younger than 60 years, 41.70% were aged 60–69 and 31.21% were 70 years and older. There were 8.65% and 14.08% of patients diagnosed with stage I and stage II disease respectively. Stage III disease was diagnosed among 36.26 % and stage IV among 29.13 % of the patients in the cohort (Table 1).

**Table 1:** Demographic and clinical characteristics of patients with gastric cancer, by diabetes status and antihyperglycemic medication use.

**Characteristics**	**Non diabetic**		**Metformin users**		**Metformin and other medication users**		**Sulphonylurea users**		**Insulin and other medication users**	
	**N**	**%**	**N**	**%**	**N**	**%**	**N**	**%**	**N**	**%**
Total	7868	100.0	143	100.0	251	100.0	115	100.0	46	100.0
Sex										
Male	4631	58.9	80	55.9	116	46.2	65	56.5	25	54.3
Female	3237	41.1	63	44.1	135	53.8	50	43.5	21	45.7
Age at diagnosis										
<60	2213	28.1	22	15.4	28	11.2	6	5.2	13	28.2
60–69	3253	41.4	64	44.7	129	51.4	50	43.5	16	34.8
70+	2402	30.5	57	39.9	94	37.4	59	51.3	17	37.0
TNM stage										
I	670	8.5	16	11.2	31	12.3	8	7.0	4	8.7
II	1109	14.1	17	11.9	36	14.3	18	15.6	6	13.0
III	933	11.8	18	12.6	23	9.2	18	15.6	8	17.4
IV	2878	36.6	45	31.4	75	29.9	40	34.8	16	34.8
Missing	2278	29.0	47	32.9	86	34.3	31	27.0	12	26.1

**Figure 2:**
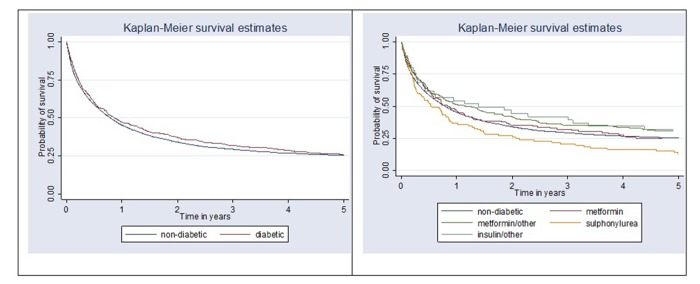
**Kaplan-Meier survival curves comparing gastric cancer-specific survival between non-diabetic and diabetic patients (p = 0.29) and by antihyperglycemic medication user group (p=0.013).**

**Figure 3:**
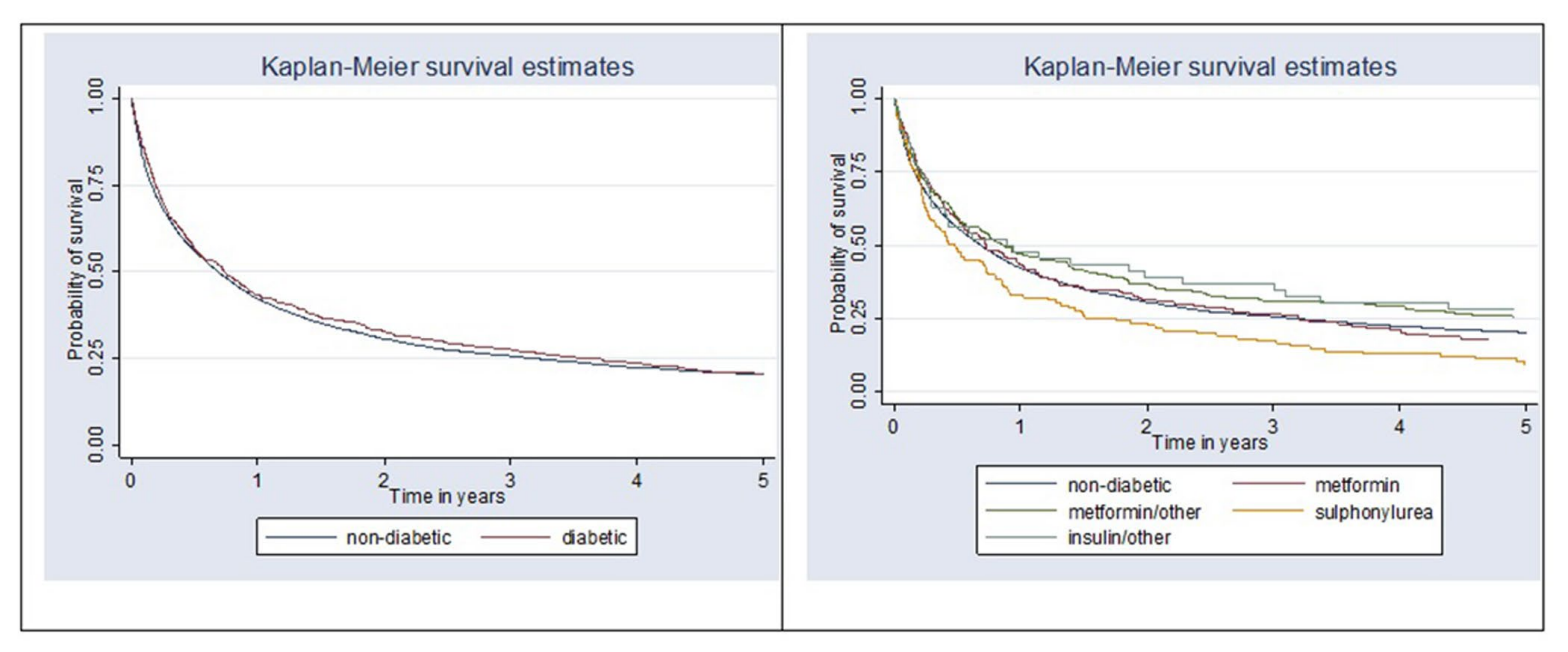
**Kaplan-Meier survival curve comparing overall survival between non-diabetic and diabetic patients (p = 0.89) and by antihyperglycemic medication user group (p = 0.052).**

Kaplan-Meier survival analysis showed no differences for gastric cancer-specific survival between non-diabetic and diabetic patient groups. Cancer-specific survival analysis by antihyperglycemic medication user groups revealed strong evidence of survival difference between groups (p = 0.013). Better survival was observed in the groups of patients using antihyperglycemic medication combinations with metformin, metformin alone or insulin. Lowest survival (and lower than in non-diabetic patients) was observed in diabetic patients who were sulphonylurea users (Figure 2). Survival analysis comparing overall survival between non-diabetic and diabetic patients (p = 0.89) showed no evidence of survival difference between groups and survival differences between antihyperglycemic medication user groups were of borderline significance (p = 0.052) Figure 3.

In the multivariate analysis after adjustment for sex, age and stage at diagnosis, there was no difference in gastric cancer-specific and overall survival between patients with diabetes compared to patients without diabetes (Tables 2). Furthermore, exposure to antihyperglycemic medication among diabetic patients did not show any death risk differences in gastric cancer-specific and overall survival (Table 3).

**Table 2:** HR and 95% CI of the association between diabetes and gastric cancer-specific mortality, and overall mortality.

**Variable**	**Gastric cancer-specific mortality**		**Overall mortality**	
	Multivariate-Adjusted HR * (95% CI)	**p-value**	Multivariate-Adjusted HR* (95% CI)	**p-value**
**Sex**				
Male	1.00	ref.	1.00	ref.
Female	0.91 (0.86–0.96)	<0.001	0.89 (0.85–0.93)	<0.001
**Age at diagnosis**				
<60	1.00	ref.	1.00	ref.
60–69	1.22 (0.15–1.30)	<0.001	1.31 (1.24–1.39)	<0.001
70+	1.83 (1.71–1.95)	<0.001	2.11 (1.98–2.24)	<0.001
**TNM stage**				
I	1.00	ref.	1.00	ref.
II	4.74 (3.93–5.71)	<0.001	2.66 (2.34–3.02)	<0.001
III	6.90 (5.73–8.31)	<0.001	3.53 (3.10–4.00)	<0.001
IV	20.31 (17.01–24.24)	<0.001	10.09 (8.97–11.35)	<0.001
**Missing**	6.44 (5.39–7.69)	<0.001	3.48 (3.10–3.92)	<0.001
**Diabetes**				
**Non-diabetic**	1.00	ref.	1.00	ref.
**Diabetic**	0.93 (0.84–1.03)	0.18	0.97 (0.88–1.06)	0.53

*Adjusted for all variables shown in table

**Table 3:** HR and 95% CI of the association between antihyperglycemic medication use and gastric cancer-specific and overall mortality.

**Cumulative dose (mg)**	Gastric cancer specific mortality HR* (95% CI)	**p-value**	Overall mortality HR* (95% CI)	**p-value**
Metformin users	1.00	ref.	1.00	ref.
Metformin and other medication users	0.91 (0.71–1.17)	0.47	0.92 (0.74–1.16)	0.49
Sulphonylurea users	1.09 (0.82–1.45)	0.06	1.02 (0.78–1.33)	0.88
Insulin and other medication users	0.85 (0.56–1.29)	0.45	0.96 (0.67–1.37)	0.11

*Adjusted for sex, age at diagnosis and stage at diagnosis

## DISCUSSION

Our current study is one of few clinical epidemiological studies to determine whether antihyperglycemic medications have a positive effect on gastric cancer survival. The results have demonstrated a null association between the effect of antihyperglycemic medication and gastric cancer survival in diabetic patients in Lithuania. However, survival analysis by antihyperglycemic medication user groups revealed strong evidence of survival differences between groups (p = 0.013). Better survival trend was observed in the combinations with metformin, metformin alone and insulin user groups. Lowest survival (and lower than in non-diabetic patients) was observed in diabetic patients who were sulphonylurea users.

It should be pointed out that overall incidence of gastric cancer and mortality from the disease are two different entities and probably linked to different factors. Only few previous studies have assessed the links between antihyperglycemic medication and survival in gastric cancer [14, 29, 30–34].

Previously Currie et al. analysed a large cohort of patients and compared patients with diabetes and cancer to patients with cancer but without diabetes. They also assessed different antihyperglycemic treatments. According to the study, compared to the nondiabetes group, mortality was increased in those on monotherapy with sulfonylureas (HR 1.13 [95% CI 1.05–1.21]) or insulin (HR 1.13 [95% CI 1.01–1.27]), but reduced in those on metformin monotherapy (HR 0.85 [95% CI 0.78–0.93]) [29]. Bowker et al. showed similar results among metformin users in all-cancer patients, and the worse results in patients on insulin or sulfonylurea monotherapy [14]. In a small prospective study Ladman et al. found that metformin use was associated with a 57% reduction in cancer-specific mortality and diabetes itself was a worse prognostic factor [30]. Similarly *van de Poll-Franse et* al showed that patients with diabetes experienced a significant increase in overall mortality (HR 1.44 [95% CI 1.40–1.49]), ranging however from 0 to 40% for different types of cancer [31]. Worse prognosis was mainly related to less aggressive cancer treatment. However, authors did not assess the effect of different antihyperglycemic medication on cancer prognosis. Tseng specifically assessed diabetes effect on gastric cancer prognosis and reported that diabetic Taiwanese had a higher risk of gastric cancer mortality and insulin had no effect on mortality in gastric cancer patients [19]. Age and male sex were associated with gastric cancer mortality, but diabetes type, insulin use, and smoking were not. Body mass index and area of residence did not show consistent association. In another large cohort form US, authors could not show the relation between diabetes and increased gastric cancer mortality (HR in men 0.99[95% CI 0.77–1.27]; HR in women 1.25[95% CI 0.90–1.73]) [32].

To our knowledge, only two recent studies assessed gastric cancer survival in diabetic patients [33, 34]. Lee et al. included 1974 gastric cancer patients with diabetes and found that metformin use was related to a statistically significant increase in overall survival, cancer specific survival and recurrence free survival compared to those who did not receive metformin (overall survival HR 0.584 [95% CI 0.369–0.926]; cancer-specific survival HR 0.57 [95% CI 0.334–0.975]; recurrence-free survival HR 0.633 [95% CI 0.410–0.977]) and metformin treatment prolonged survival in diabetic patients to a rate comparable to that in non-diabetic patients. The cumulative use of metformin was shown to reduce the risk of recurrence, all-cause mortality, and cancer-specific mortality as well [33]. Last year, similarly to our study, Baglia et al. showed significantly worse survival results in diabetic patients with gastric cancer using sulfonylurea (HR 2.05 [95% CI 1.09–3.84]) or insulin (HR 1.45 [95% CI, 0.99–2.10]) and no effect with metformin use (HR 1.01 [95% CI 0.48–2.12]) [34]. Although we could not find significant differences between the antihyperglycemic medication groups and gastric cancer survival, we did see a tendency towards worse results in patients on sulfonylurea. Differently from two studies mentioned above, we assessed patients with gastric cancer only and the effect of most often used antidiabetic drugs (metformin, sulfonylurea and insulin).

The present study has several advantages: it has a large sample size, which reduces the likelihood of random error; includes the entire regional population, thus minimizing selection bias; and it does not rely on self-reports.

Our study has some limitations as well. First of all, we could not evaluate confounding factors, including body mass index, obesity, smoking history, lifestyle, dietary habits, and *Helicobacter pylori* (*H. pylori*) infection as this data was unavailable. Finally, we did not directly measure insulin levels or insulin resistance, therefore, the linkage between hyperinsulinemia and cancer incidence could only be inferred from the drug-cancer associations.

To conclude, our study did not show any relation between diabetes, antihyperglycemic medication use and gastric cancer survival. Further randomized controlled studies are needed to determine the effect of antihyperglycemic meadication (mainly metformin) use on gastric cancer risk and survival.

## MATERIALS AND METHODS

### Vilnius regional biomedical research ethics committee approved the study

All data used in this study was provided by The Lithuanian Cancer Registry and comprised individual cancer patient records which were linked to The National Health Insurance Fund (NHIF) database. NHIF collects demographic data and entries on the primary and secondary healthcare services provided, emergency and hospital admissions, and prescriptions of reimbursed medications.

Using the Cancer Registry, we identified all patients with newly diagnosed primary invasive gastric cancer (ICD-10 code C16) between January 1, 2003 and December 31, 2013. Information regarding the diagnosis of T2DM (ICD-10 code E11) and diabetes treatment were obtained from NHIF.

Patients were excluded from the cohort if they were identified as death certificated only cases, patients with malignancy prior to gastric cancer and cases with diabetes diagnosed after the diagnosis of gastric cancer. A flow chart outlining the selection of the cohort is presented in Figure 1.

### Exposure definition

Cohort members were classified into five groups: four groups of T2DM (referred to as diabetes in this study) patients according to treatment: metformin users; metformin and other medication users; sulphonylurea users; insulin and other medication (except for metformin) users; and non-diabetic group.

The diabetes group was restricted to patients who had used antihyperglycemic medication. This latter restriction was necessary to ensure that all patients actually had diabetes. Patients not reported with a diabetes diagnosis in the NHIF database were classified as non-diabetic. Exposure to antihyperglycemic medication was identified from linked prescription data. Exposure (yes/no) was defined according to whether or not the individual had a supply of antihyperglycemic medication available at any point.

### Outcomes

The primary outcome was gastric cancer-specific survival; overall survival was also examined in secondary analysis. Cohort entry corresponded to the date of the gastric cancer diagnosis. Patients were followed-up with respect to vital status until December 31, 2017.

In this analysis, survival outcomes in patients with gastric cancer were compared between non-diabetic and diabetic patients and by antihyperglycemic medication user groups. Gastric cancer-specific survival was the primary outcome, measured from the date of gastric cancer diagnosis to date of death due to gastric cancer, or last known date alive. Patients who were not deceased or who died of causes other than gastric cancer were censored at the last known date alive or date of death, respectively. Overall survival was analysed as a secondary outcome, and defined as the period from the date of diagnosis of gastric cancer to the date of death or last known date alive. For this secondary outcome, only those patients who were not deceased were censored at the last known date alive.

### Statistical analysis

For the analyses, patients were categorized by sex, age at diagnosis (<59, 60–69 and 70+ years), and stage at diagnosis (TNM classification).

Patient demographic and clinical characteristics were tabulated for the all exposure groups as described above. Kaplan-Meier survival analyses stratified by exposure group were used to generate median survival curves for both gastric cancer-specific and overall survival. Survival curves were compared using the log-rank test.

Cox proportional hazard models were used to estimate hazard ratios (HR) with 95% confidence intervals (CI) for associations between diabetes status, antihyperglycemic medication exposure and gastric cancer-specific survival and overall survival. Multivariate Cox proportional hazard models for gastric cancer-specific and overall survival included known prognostic factors such as sex, age at diagnosis, and stage at diagnosis.

All statistical analyses were carried out using STATA 11 statistical software (StataCorp. 2009. Stata Statistical Software: Release 11.0. College Station, TX, USA).
